# Designing and implementing a skills program Using a clinically integrated, multi-professional approach: Using evaluation to drive curriculum change

**DOI:** 10.3885/meo.2009.F0000221

**Published:** 2009-09-20

**Authors:** Sandra E. Carr, Antonio Celenza, Fiona Lake

**Affiliations:** * Education Centre, Faculty of Medicine, Dentistry and Health Sciences, University of Western Australia; † Discipline of Emergency Medicine, University of Western Australia and Sir Charles Gairdner Hospital; ‡ School of Medicine and Pharmacology, Royal Perth Hospital, University of Western Australia

**Keywords:** multi-professional, skills training, undergraduate medicine

## Abstract

The essential procedural skills that newly graduated doctors require are rarely defined, do not take into account pre-vocational employer expectations, and differ between Universities. This paper describes how one Faculty used local evaluation data to drive curriculum change and implement a clinically integrated, multi-professional skills program. A curriculum restructure included a review of all undergraduate procedural skills training by academic staff and clinical departments, resulting in a curriculum skills map. Undergraduate training was then linked with postgraduate expectations using the Delphi process to identify the skills requiring structured standardised training. The skills program was designed and implemented without a dedicated simulation center. This paper shows the benefits of an alternate model in which clinical integration of training and multi-professional collaboration encouraged broad ownership of a program and, in turn, impacted the clinical experience obtained.

Undergraduate medical curricula should train graduates to perform essential procedural skills since many of these skills will be performed unsupervised upon commencement of work.^1^ In many jurisdictions, medical councils have described the expected competencies of junior doctors, examples being the “The Australian Curriculum Framework for Junior Doctors” developed by the Confederation of Postgraduate Medical Education Councils in Australia and “The New Doctor 2007” from the General Medical Council in the United Kingdom (UK).^2^,^3^ These guidelines describe what should be learned during the early postgraduate years but do not address what should have been learned during undergraduate training which remains the domain of universities.

It is recognized that ensuring students’ preparedness to practice as a doctor at graduation is difficult^4^–^6^ and students often feel unprepared in the performance of procedural skills.^7^ The essential procedural skills that newly-graduated doctors should perform competently are rarely defined,^8^ do not take into account pre-vocational employer expectations, and differ between Universities. In some places, only 75% of the intended skills based curriculum is being learned.^9^ Even if they have been formally taught, many students often have not had adequate practice.^10^,^11^ Ethical and medico-legal changes, patient safety concerns and increasing patient medical knowledge and expectations make it difficult for students to learn and practice procedures on patients.^12^–^14^ Increasing student numbers, decreasing clinical teaching resources and fewer clinical opportunities are also resulting in less experience for students.^15^–^18^ Some medical schools have addressed these concerns by focused curricular change, introducing simulation centers that ensure adequate teaching of skills and logbook or portfolios to record adequate practice and skills based assessments to ensure competence.^14^,^19^–^22^

In our experience, the multiple disciplines contributing to an undergraduate medical curriculum and the hospitals that employ graduates have diverse expectations regarding procedural skills that are deemed essential for graduates. This results in disagreement on what should be taught, variation or duplication in teaching, inefficiencies and confusion, all of which affects students’ learning experiences and opportunities. Despite a medical curriculum redevelopment at the University of Western Australia (UWA), evaluations continued to demonstrate graduates’ limited preparedness in performing procedural skills. Curriculum review showed a poorly coordinated approach to teaching procedural skills. This paper describes how curriculum change occurred, resulting in the implementation of a clinically integrated skills program at UWA, and discusses how this model of training may have an impact on graduates’ preparedness to practice.

The plan was to involve all relevant groups (across disciplines, professions and levels of training) to plan and implement a program that would teach core skills in a standardised fashion, encourage practice in the clinical setting, and integrate with prevocational training. The program had to be sustainable and supportable by the usual running budget for clinical teaching.

## Processes and Results

**Setting** – The medical course at UWA is a 6-year undergraduate course with approximately 120–150 students per year. Most postgraduate year 1 (PGY1) doctors in the teaching hospitals of Western Australia are UWA graduates. The medical school curriculum redesign commenced in 1998 with graded implementation beginning in 2000.

**Review of curriculum** – During the curriculum restructuring, one of the themes reviewed was resuscitation training. Initially, a small working party representing surgery, anaesthesia, intensive care, emergency medicine and students convened to identify needs and plan coordination of resuscitation teaching. This led to a review of all procedural skills teaching and a document titled “Core Curriculum in Clinical Practice”, describing the skills, clinical presentations and conditions new graduates should have learned and practiced. A broad list of skills from the Core Curriculum in Clinical Practice was defined and further reviewed by a modified Delphi approach by academic staff from the departments of paediatrics, medicine (including anaesthetics), surgery (including emergency medicine and other surgical specialties), women's health and general practice to form a “list of proposed skills”.

**Analysis of needs by postgraduate groups** – The next step was to link the undergraduate-level expectations with postgraduate expectations. Through a structured questionnaire, members of the postgraduate medical education committees of hospitals that provided employment to graduates from UWA, along with members of the prevocational training committee of the local medical board, were asked to select ten skills from the proposed skills list they deemed essential for doctors to have at graduation (Table 1). For each of the ten selected skills respondents were asked to indicate:

- When, where and to what levels commencing postgraduate Year 1 (PGY1) doctors should be trained
- Current teaching and whether this was considered adequate
- Deficiencies in teaching staff or training resources
- Optimal teaching and resources
- The need for formal assessment and reaccreditation.

**Table 1.** Initial List of Procedural Skills derived from the Core Curriculum in Clinical Practice

**Procedural Skills**
Venipuncture ECG PEFR, Spirometry MSU, Urinalysis, Pregnancy test Finger prick glucose Collection of swabs and slide smear Use of standard precautions Vaginal pH testing Gown and gloving 10. Drug administration by sc, im, iv routes Local anesthetic infiltration Wound closure, Removal of sutures Aseptic dressing change Urinary catheter insertion (male and female) Bandaging and splinting of limbs Limb back slab plaster Removal of plaster Basic life support (airway positioning, management of choking, 1 & 2 person CPR, positioning of unconscious patient) Defibrillation/advanced life support Spinal immobilization Use of airway adjuncts and suctioning Manual ventilation with bag-mask-valve Endotracheal intubation Oxygen and nebulizer therapy Intravenous cannula insertion Setting up iv infusion Fluid and blood component therapy Managing a pediatric airway Test visual acuity including use of pinhole Lid eversion Insertion of eye drops and padding Syringe an ear Use of nasal speculum Direct and indirect laryngoscopy Pap smear and swab collection Initiate neonatal resuscitation

Twenty of the 50 questionnaires distributed were returned (response rate= 40%). Of the skills on the list, 21 were considered to be essential by at least one respondent. The most highly rated skills were identified as essential by between 50 and 95% of respondents. The other skills were identified as essential by 35% or less of respondents. Two additional skills (inter-costal catheter and lumbar puncture) were suggested. This resulted in a final short-list of skills in which the postgraduate clinicians felt doctors should be competent in on the first day of work.

The top skills depicted in Table 2 included the ability to resuscitate and practice emergency interventions, insert and manage intravenous therapy, monitor and interpret vital signs in a sick patient, manage oxygen therapy, insert a urinary catheter, interpret arterial blood gas results, and suture a simple wound. It was felt these skills should be assessed but only life support skills required reaccreditation. Ninety percent of respondents expected the PGY1 doctor to be able to practice these skills unsupervised on day one of working.

**Table 2.** Essential skills identified by clinicians responsible for postgraduate training When should training occur, what is the current and the optimal teaching, and is assessment required?

**Skill**	**Start of PGY1 (% agree)**	**End of PGY1 (% agree)**	**Independent Practice**	**Current Teaching (ad hoc/structured)**	**Optimal Teaching (ad hoc/structured)**	**Formal Assessment (yes/no)**
**ECG – performing and basic interpretation**	53%	42%	(47%)	Ad hoc	Structured	No
**Intravenous therapy including intravenous cannula insertion, setting up iv infusions, administration of iv drugs, fluid therapy and fluid charts**	79%	21%	(68%)	Ad hoc	Structured	Undecided
**Senior First Aid including recognition of danger, airway positioning, choking, 1 & 2 person CPR, positioning of unconscious patient**	94%	0%	(78%)	Structured	Structured	Yes
**Airway management including use of adjuncts and manual ventilation**	65%	29%	(71%)	Structured	Structured	Yes
**Monitoring and interpreting BP, P and R in a sick patient**	69%	25%	(56%)	Ad hoc	Structured	No
**Oxygen therapy including nebuliser therapy, use of pulse oximetry**	80%	7%	(73%)	Ad hoc	Structured	No
**Urinary catheter insertion (male and female)**	50%	43%	(64%)	Ad hoc	Structured	Yes
**Defibrillation**	58%	42%	(83%)	Structured	Structured	Yes
**Arterial puncture and performing and interpreting arterial blood gas sampling**	9%	91%	(82%)	Ad hoc	Structured	No
**Wound management, including simple wound closure and removal of sutures**	50%	40%	(50%)	Ad hoc	Structured	Yes

After discussion, some essential skills (i.e., interpretation of arterial blood gases and the arterial puncture) were thought to require theoretical knowledge and understanding of application at an undergraduate level but the need to apply the practical skill would not be required until completion of the PGY1; these skills were not put forward for inclusion in the training program. Similarly, even though not rated by the clinicians, patient handling skills (moving patients safely) were deemed essential by the skills working party and therefore were included in the training program.

**Implementation** – A curriculum skills map was created that described the essential and desirable skills and the current learning experiences. Methods of teaching used, level of skill expected, assessment strategies and whether practise in the clinical setting was encouraged or available were included.

A Skills Working Party (SWP) with representatives from several disciplines (medicine, surgery, pediatrics, emergency medicine, women's health) and a range of health professions used the short-list and the curriculum skills map to develop a centrally coordinated skills training program. The program aimed to ensure all students were taught the basic required skills in a standardised fashion and, using assessment as a driver, to make use of clinical opportunities when they arose. Once the program had been developed, further input was sought from a broader range of different health professionals who were asked to comment on the learning outcomes, methods and proposed assessment strategies and tools. These health professionals were then approached to deliver the training. For example, nurses specifically trained to manage continence were asked to teach the urinary catheterisation workshops; phlebotomists from all of the local teaching hospitals agreed to provide the venipuncture training; physiotherapists conducted the manual handling (moving patients safely) sessions.

Implementation commenced across all clinical years in 2004 and included the following elements:

- Pre-requisite for course entry: A Senior First Aid certificate was required to be obtained by students prior to the end of 1^st^ year.
- Year 4: During the first 2 weeks of Year 4, the first entirely clinically-based year, students participate in standardized simulation workshops of 2–4 hours in groups of 6 to 8 students. The workshops are followed by facilitated opportunities to practice in clinical settings through ward placements, nursing attachments, phlebotomy services, and continence nurses. The learning outcomes are summarized in Table 3. An example of a specific skill outcome is shown in Table 4. All students received formative assessment with written and verbal feedback at the end of each simulation workshop, covering all components of performing the skill. The skills workshops were taught by a range of health professionals (nurses, phlebotomists, physiotherapists, continence advisors, infection control personnel and doctors) from disciplines within the Faculty and external service providers. A suturing workshop was conducted during the clinical attachment in Surgery. During the remainder of the academic year, students recorded their experiences performing these procedural skills, with supervising practitioners (doctors, nurses, phlebotomists) encouraged to provide feedback on observed performance using criterion-referenced assessment detailed in a logbook. The logbook is submitted at the end of the academic year. All of these procedural skills are assessable in the end-of-year OSCE.
- Year 5: Neonatal resuscitation and paediatric resuscitation skills, together with a refresher of adult resuscitation, are taught in small groups using low-fidelity simulation.
- Year 6: Workshops are conducted to train basic airway management (Rural General Practice), suturing (Surgery), limb splinting and plastering (Emergency Medicine). Students need to attend and be assessed as competent in an Immediate Life Support Course, accredited by the Australian and UK Resuscitation Councils.

**Table 3.** The Learning Outcomes for Year 4 Skills Training Program

At the end of the program the students are expected to: Discuss issues of confidentiality and legal requirements when obtaining consent to perform a procedure. Describe and demonstrate principles of asepsis through hand washing, and preparation and maintenance of a sterile field when performing the stated skills. Describe and demonstrate ability to correctly perform the following skills with supervision: Phlebotomy Injections (subcutaneous (SC), intravenous (IV) and intramuscular (IM)). Intravenous (IV) cannulation Urinary catheter insertion Principles of Manual Handling Cardiopulmonary resuscitation (CPR)-Describe and demonstrate the basic life support algorithms, use of airways and bag-mask ventilation and shock advisory defibrillation. Discuss with clinical insight when these procedural skills would be required.

**Table 4.** Outcomes to be achieved in the Year 4 urinary catheterization workshop, workshop process, and assessment

**Learning Outcomes**	**Workshop Process**
State reasons for urinary catheterisation Correctly insert urinary catheter for male and female Explain procedure/obtain consent/maintain privacy Prepare equipment/select the correct catheter size/type Maintain asepsis Don sterile gloves correctly Avoid contamination of sterile field Take appropriate action if sterile field is contaminated Insert catheter correctly (male and female) Avoid inflating catheter balloon whilst still In urethra Recognise situations which require expert help Recognise allergic reaction to lignocaine gel Collect specimen if required Connect drainage bag Dispose of equipment correctly Record Patient Clinical Status-document urinary output	In groups of 12, students attend a one-hour group discussion with two Continence advisors. They discuss the anatomy, clinical decision making and practice related to urinary catheterisation and observe a demonstration of the procedure. Then over two hours, in groups of 6 students with 1 instructor, students prepare for and perform urinary catheterisation on both male and female mannequins under direct observation.
	**Formative Assessment**
	Students are given direction with their first attempt and observed performing the procedure using the outcomes as criteria. Students are rated as either having demonstrated or not demonstrated the skill.

Funding was diverted from all disciplines that taught in the clinical years to pay a coordinator and part-time teaching staff. Funding from a competitive grant paid for physical teaching resources, such as manikins, that were used in the workshops. The resources were also made available for loan to all disciplines throughout the year.

**Evaluation of the Program** – A participatory evaluation process was used to evaluate the skills training processes and the effect of the Year 4 program on clinical experience.^23^,^24^ A survey of self-assessed ability to perform the skills was collected from the Year 5 and 6 participants immediately before and at the beginning of each subsequent academic year (2004 to 2006). A Wilcoxon sign rank test was used to evaluate for shifts in this self-rated ability.

In addition, a survey focusing on the key Year 4 Workshop skills was administered before and 4 weeks after the training workshops. Using a five-point Likert scale (5= Very Well, 4= Well, 3 = Average, 2= Poorly, 1= Very poorly), students evaluated their preparedness to complete each of the procedures listed on the annual survey. Students also documented the amount of experience they had obtained performing procedures during the preceding 12 months. The amount of experience was confirmed through the collected logbooks. Results from students receiving the formal skills program were compared to the previous cohort (2003) who had not received structured skills training but had the same clinical attachments. A one-way analysis of variance with the Bonferroni post-hoc multiple comparisons correction was used to test for significant differences between the intervention and comparison group. Additionally, at the end of each skills training workshop, students were formatively assessed to ensure they could perform the procedure correctly in a simulated setting, could discuss with clinical insight when the procedures would be performed. They were also asked for their formative evaluation of the workshop. Students were not assessed on their ability to obtain consent from patients, although informed consent was covered explicitly on other areas of the course. The multi-professional group of trainers reviewed and discussed the evaluation results as a group to refine the delivery of training for the subsequent year.

When looking at the impact of the Year 4 workshops, the majority of students met the required standard on the first attempt in the formative assessment; all students met the criteria on their subsequent attempt. There was a significant improvement in self-rated ability in all skills 4 weeks after the workshops (p < 0.01) (Table 5). Evaluation of the processes surrounding the skills workshops was positive, with a high level of agreement that they achieved their intended outcomes. When compared with the preceding cohort who did not receive the skills training, self-rated preparedness at the commencement of Year 5 to perform skills was significantly higher for four of the skills: injections, inserting intravenous cannula, phlebotomy and performing cardio-pulmonary resuscitation (Table 6).

**Table 5.** Student self rated ability before and 4 weeks after skills training

Response scale = 1 (strongly disagree) to 5 (strongly agree).					

	**Before N = 119 (100%)**		**After (4 weeks)**	
			
**Procedure**	**n**	**Median (IQR)**	**n**	**Median (IQR)**	**Sign Rank (p) Difference**
**Use principles of asepsis**	119	3 (3,4)	85	4 (4,4)	0.001^*^
**Phlebotomy**	119	2 (1,4)	85	4 (4,4)	0.001^*^
**IV, IM, SC injection**	119	2 (1,4)	85	4 (4,4)	0.001^*^
**IV Cannula**	119	2 (1,5)	85	3 (2,4)	0.012^*^
**Insert a urinary catheter**	118	2 (1,5)	85	3 (2,4)	0.008^*^
**Demonstrate 1 & 2 person CPR**	118	3 (2,4)	85	4 (4,4)	0.001^*^
**Manual handling(Move patients safely)**	119	2 (1,4)	85	4 (4,4)	0.021^*^

^*^Wilcoxon Signed Rank Test, significant at p < 0.05.

Note: 85 of the original 119 (71.4%) students completed the follow up survey 4 weeks after training.

**Table 6.** Impact of skills training program on students’ self-rated ability to perform skills measured after skills training and 12 months clinical experience, compared to 12 months clinical experience and no training.

**Skill**		**N**	**% well prepared**	**p**
**Use aseptic technique**	No Training (2003)	89	26	0.303
	Trained (2004)	112	34	
**Phlebotomy**	No Training	89	52	0.001*
	Trained	112	77	
**IM, IV and SC injections**	No Training	89	37	0.001*
	Trained	112	57	
**IV cannula**	No Training	89	35	<0.001*
	Trained	112	55	
**Urinary catheter**	No Training	89	10	0.425
	Trained	108	19	
**Demonstrate CPR**	No Training	89	21	0.002*
	Trained	109	38	

^*^Mann Whitney U test, significant at p < 0.05.

It appears the workshops and logbook may have had an impact on practice during clinical attachments, with significantly greater experience in some procedures being gained, as compared to a previous cohort that did not receive a formal training program (Table 7). The impact of the skills training on students’ confidence and clinical experience was also shown over the longer term through the higher ratings of self-rated preparedness in clinical and procedural skills.

**Table 7.** Impact of skills training compared with no skills training on the number of procedures performed over 12 months as determined from logbook records

		**No Training**			**Training**		
					
Skill	n^*^ (of 124)	**Mean number of procedures (95% CI)**	**% of students never performed skill**	n^*^ (of 125)	**Mean number of procedures (95% CI)**	**% of students never performed skill**	**p value**
**Use aseptic technique**	73	2.9 (2.5,3.4)	12	114	3.3 (2.9,3.7)	3	p = 0.229
**Phlebotomy**	73	4.4 (4.1,4.7)	1	115	4.8 (4.7,5.0)	0	P = 0.011^†^
**IV cannula**	72	3.3 (2.84, 3.7)	10	111	3.9 (3.6,4.2)	3	p = 0.017
**Urinary catheter- male**	73	1.1 (0.8,1.4)	38	84	1.4 (1.1,1.5)	27	p=.258
**Urinary catheter—female**	73	0.56 (0.3, 0.8)	64	64	1.2 (.9,1.4)	42	P = 0.003^†^

^*^n = number who completed the logbook.

^†^Significant with Bonferroni correction at 0.01.

## Discussion

Undergraduate medical curricula must train graduates to perform essential procedures so they are ready to commence practice. This paper has described how one Faculty used local evaluation information to demonstrate graduates’ lack of preparedness to perform essential skills and to convince staff of the need for structured, standardised skills training and the linking of undergraduate training with postgraduate needs. The results of program evaluation indicated that the workshops’ processes and format have been valued by students, have enhanced student ability and preparedness to perform several of the procedural skills and also increased the amount of clinical experience they obtained. While relying on self-ratings of preparedness as a key evaluation measure may be a limitation, others have shown a link between poor perceived preparedness and limited exposure to clinical experience.^25^ Additionally, while students are supervised when performing procedures, introducing structured summative assessment has proved difficult, thus limiting outcome evaluation of the program. Longer term summative evaluation is also being conducted to measure retention of resuscitation skills into internship. The results of this study are not as yet available.

Much of the literature related to skills training evaluates a single procedure or the utility of skills centers and simulation. This paper showed the benefits of another model in which the skills training is offered adjacent to and linked with clinical attachments by the health professionals’ students work with and alongside. Interdisciplinary participation encouraged broad ownership and in turn impacted clinical experience, with a wider group of professionals than just doctors supporting students’ learning. This multidisciplinary program increased dialogue across clinical sites, for example, between nurses and doctors responsible for infection control and procedures, and between undergraduate and prevocational trainers, with better agreement on what should be taught, when, and how.

During this 3-year process, several key factors have been highlighted for implementing a successful and sustainable skills training program. Consulting widely with local clinicians and sharing the results of consultation openly is important but takes time. We required over six months for consulting and establishing arguments for implementing the training program. In the planning and implementation phase, choosing working party representatives with care (those already using simulation or staff ready for change) was important to ensure a willing partner within relevant disciplines.

Outside of the undergraduate course, we felt it was important to engage postgraduate educators, so they agreed on skills covered at an undergraduate level and better understood what training and assessment had occurred. There is often duplication in teaching, to the annoyance of students or graduates or teachers. Vertical integration is now occurring, as many skills, both clinical and procedural, require ongoing development.

With significant movement of students across Australian states and now with most Australian states having more than 1 medical school, some general agreement about what should be taught, when it should be taught and how, particularly with procedural skills, is useful. The idea of a national exit examination has been raised,^26^ an element that would drive such a development.

Within the medical school, the use of ‘central funding’ so no one group is disadvantaged or favored was important, as was ensuring an ongoing source of funding that was not grant based. This program had to be established and run without a substantial injection of funds and without the luxury of a dedicated skills center. We obtained a one-time grant to buy equipment and decided to establish an “Equipment Library”. Any member of Faculty can borrow the part-task trainers purchased with the funding and use them at no cost year round. This minimises the number of part-task trainers that are gathering dust in cupboards in different schools. We also accessed disposable equipment from a range of sources, such as free or expired disposable equipment from pharmacy, theater, labor ward, etc. which has meant training costs have been kept to a minimum.

As the program was cross-disciplinary, we wanted to keep it out of the usual School structure. Therefore an individual able to work with different health professionals took responsibility for coordinating the training workshops’ development, recruiting and training the trainers, and liaising between the Faculty, Schools within the Faculty and the health care providers where students are placed clinically. In addition, this person worked with clinical staff from the hospitals so that teaching was based on current practice and so that trainers were more comfortable supervising and giving feedback to students in the clinical setting.

We felt it important that teaching was encouraged to carry over into clinical attachments. Though students are expected to be able to perform the procedure correctly and safely at the end of a workshop, they need practice to perform the skills consistently when not observed, and they need to perform them in a real context.^27^ In addition, what was learned in workshops required the students’ self direction to apply the skills elsewhere, something which does not automatically happen if not structured into the program.^28^ Both the logbook, an assessment requirement, and knowing that the material could be included in the end of year clinical examination appeared to have an impact. Most skills were performed more often and a smaller number of students reported never performing a skill as compared to students who had not received training or encouragement to practice through opportunities arising in routine clinical work. However, even after training a substantial number of students had still never performed male or female catheterisation, a procedure which would be performed in hospitals relatively often. This procedure is performed mainly by nurses, with doctors often not being called unless there is a problem; this presents a challenge if the student has had limited prior experience. Making a certain number of procedures mandatory and offering particular attachments (surgery, spinal units) where procedures are performed often may address this deficit.

One of the strengths of our approach has been to bring together different health professions, clinical departments and pre-vocational organisations so that a sense of skills-training ownership remains based within the clinical areas. This gives the students the best opportunities for ongoing experience and allows them to learn from those who will be their clinical colleagues when they begin work as junior doctors.
